# Role of advanced imaging in COVID-19 cardiovascular complications

**DOI:** 10.1186/s13244-021-00973-z

**Published:** 2021-02-24

**Authors:** Federica Catapano, Livia Marchitelli, Giulia Cundari, Francesco Cilia, Giuseppe Mancuso, Giacomo Pambianchi, Nicola Galea, Paolo Ricci, Carlo Catalano, Marco Francone

**Affiliations:** 1 Department of Radiological, Oncological and Pathological Sciences, “Sapienza” University of Rome, Viale del Policlinico 155, 00161 Rome, Italy; 2 Department of Experimental Medicine, “Sapienza” University of Rome, Viale del Policlinico 155, 00161 Rome, Italy; 3 Unit of Emergency Radiology, Policlinico Umberto I, Policlinico Umberto I, Sapienza University of Rome, Viale del Policlinico 155, 00161 Rome, Italy

**Keywords:** COVID-19, CMR, CT, Cardiac injury, Pulmonary embolism

## Abstract

Clinical manifestations of COVID-19 patients are dominated by respiratory symptoms, but cardiac complications are commonly observed and associated with increased morbidity and mortality. Underlying pathological mechanisms of cardiac injury are still not entirely elucidated, likely depending on a combination of direct viral damage with an uncontrolled immune activation. Cardiac involvement in these patients ranges from a subtle myocardial injury to cardiogenic shock. Advanced cardiac imaging plays a key role in discriminating the broad spectrum of differential diagnoses. Present article aims to review the value of advanced multimodality imaging in patients with suspected SARS-CoV-2-related cardiovascular involvement and its essential role in risk stratification and tailored treatment strategies. Based on our experience, we also sought to suggest possible diagnostic algorithms for the rationale utilization of advanced imaging tools, such as cardiac CT and CMR, avoiding unnecessary examinations and diagnostic delays.

## Key points

1. Cardiac involvement is common in COVID-19 patients, leading to a morbidity/mortality increase.
2. Cardiac complication includes, among others, myocarditis, acute coronary syndrome and thromboembolic events.
3. Advanced imaging plays a key role in differential diagnosis of cardiac manifestations.

## Introduction

Coronavirus disease 2019 (COVID-19) is a pandemic disease caused by a novel single-stranded enveloped RNA virus, severe acute respiratory syndrome coronavirus 2 (SARS-CoV-2), the 7th known human coronavirus.

The virus enters cells through the angiotensin-converting enzyme 2 (ACE2) receptor, mostly expressed in lung alveolar cells, cardiac myocytes and vascular endothelial cells.

Clinical manifestations of COVID-19 are dominated by respiratory symptoms, due to the tropism of the SARS-CoV-2 for the lungs, where it causes interstitial pneumonia [1]. The most common severe complications are acute respiratory disease syndrome (ARDS) and systemic inflammatory response syndrome (SIRS), which can lead to multiorgan failure (MOF) and shock.

Both direct and indirect involvement of other organs is common, and, although the underlying pathological mechanism is still under investigation, the cardiovascular system seems to be particularly affected.

Cardiac injury was early recognized among COVID-19 cases in China; in the report from the National Health Commission almost 11.8% of patients without underlying cardiovascular disease had cardiac injury during hospitalization, showed by elevated T-troponin (TnT) levels and/or new onset of ECG/echocardiographic abnormalities [2].

In addition to its prevalence, cardiac injury seems to be significantly associated with fatal outcome.

Retrospective studies among hospitalized COVID-19 patients have reported a more severe respiratory disease in patients with cardiac injury, requiring in greater proportion both noninvasive and invasive mechanical ventilation [3], and markedly higher mortality rate [3, 4].

Several case reports and case series released so far describe cardiovascular manifestations among COVID-19 patients, such as myocarditis [5, 6], acute coronary syndrome (ACS) [7], arrhythmias, pericarditis [8] and venous thromboembolic events.

Along with the understanding of the underlying pathological mechanisms of myocardial injury, a prompt diagnosis becomes essential for risk stratification and to define tailored treatment strategies.

Present article aimed to review the role of advanced multimodality imaging in patients with suspected SARS-CoV-2-related cardiovascular involvement.

Current emerging scientific evidence has been combined with our 8-month clinical work as one of the national referrals for COVID-19 infection. Based on our experience, we also sought to suggest possible diagnostic algorithms for the rationale utilization of advanced imaging tools in this specific clinical setting.

### Pathophysiology of myocardial injury

Although underlying mechanism leading to cardiac injury in COVID-19 patients is not entirely understood, several potential pathophysiological pathways have been proposed [9, 10].

Most likely scenario comprehends a multifactorial etiology based on a synergic effect of several mechanism, both direct or indirect, including:

- the so-called “cytokine storm,” whereby an uncontrolled immune cells activation leads to overproduction of pro-inflammatory cytokines, with disruptive consequences ranging from high fever to vascular malfunction, which can lead to inadequate organ blood supply, resulting in MOF. The increase levels of reactive oxygen species (ROS), caused by the pro-inflammatory state, result in endothelial dysfunction, which plays a pivotal role in the genesis of hypertension, atherosclerosis and other cardiovascular diseases (CVD);
- direct cytotoxic effects on interstitial cells or macrophages within cardiac tissue, hypothesis supported by viral genome detection within the myocardium in recent autoptic studies [11];
- potential downregulation of ACE2 expression in the heart, as demonstrated in a murine model of SARS-CoV-2 infection by Oudit et al. [12]. ACE2 seems to provide a protective effect on cardiovascular system, through several mechanism, including anti-inflammation, anti-fibrosis, anti-oxidation, and vasodilation [13]. Therefore, ACE2 under expression in COVID-19 patients is supposed to lead to cardiac dysfunction and progression of atherosclerosis;
- low oxygen blood levels, detected in this cohort of patients as a consequence of pulmonary disfunction, can cause an insufficient energy intake to cardiomyocyte, increasing anaerobic fermentation. Accordingly, intracellular acidosis and ROS production destroy the cell membrane. Moreover, hypoxia can lead to intracellular influx of calcium ions, contributing apoptosis of cardiomyocytes [14];
- collateral effects of several drugs widely used in COVID-19 patients, such as antiretroviral therapy, azithromycin and tocilizumab. Indeed, these drugs could lead to electrophysiological alteration as well as be involved in drug–drug interaction with some cardiovascular treatments.

**Fig. 1:**
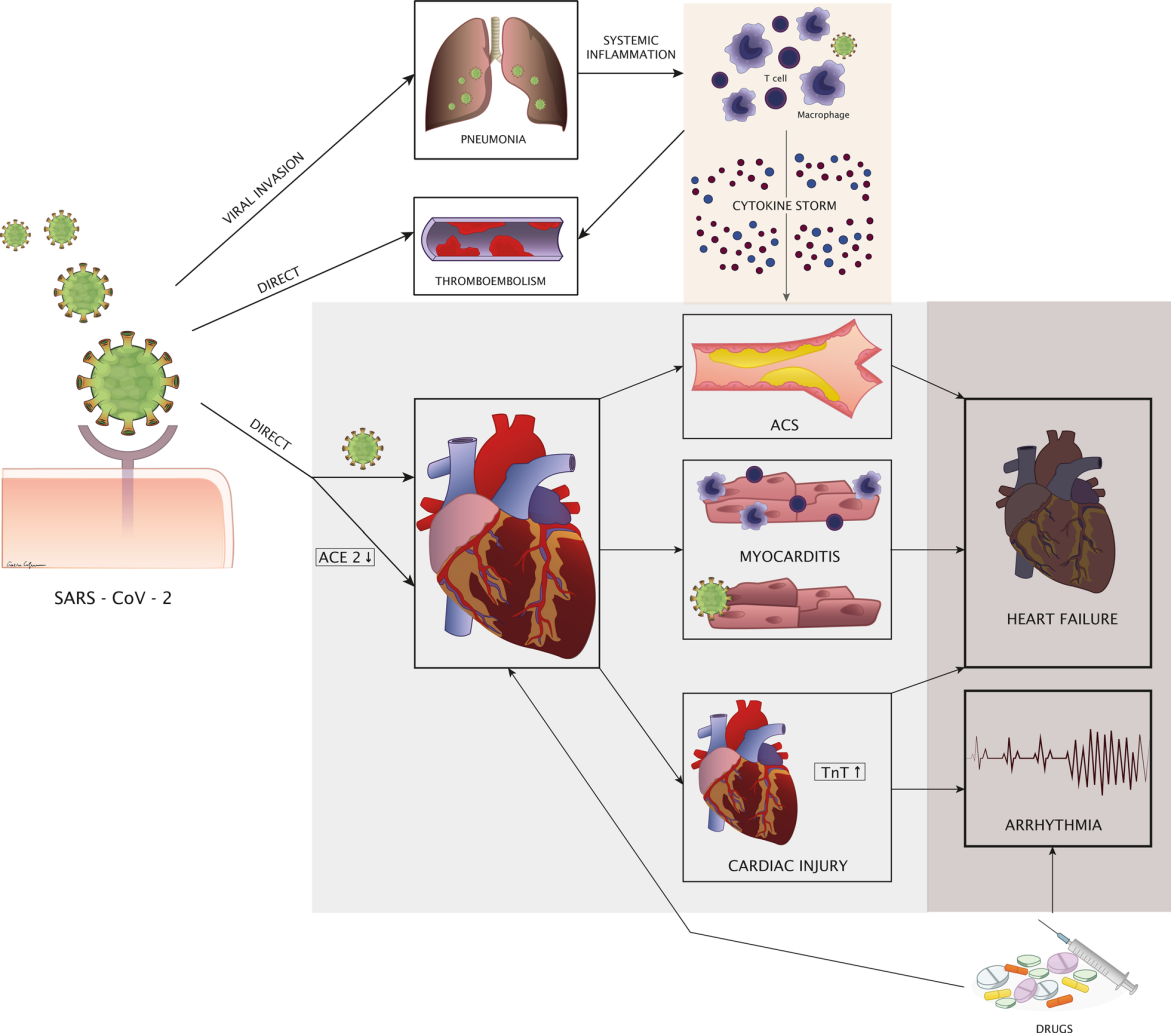
Hypothetical pathophysiology patterns concurring in cardiovascular involvement in COVID-19. SARS-CoV-2 enters cells via ACE2 receptor in type 2 in pneumocytes, endothelial cells, pericytes and cardiac myocytes, causing direct damage. Systemic inflammation and uncontrolled immune cell activation lead to a ‘cytokine storm’ which can contribute to destabilize atherosclerotic plaques and potentially trigger the onset of myocarditis trough T cells and macrophages infiltrations. Direct viral cardiac injury can provoke the development of arrhythmias, as well as several medications used in COVID-19 patients

Possible pathogenesis of myocardial involvement is illustrated in Fig. 1.

From this background emerges clearly that SARS-CoV-2 infection is associated with a wide spectrum of cardiovascular (CV) manifestation, ranging from a subtle myocardial injury to cardiogenic shock.

Overall, pneumonia, older age, pre-existing cardiovascular diseases and greater severity of the disease at presentation lead to an increased risk of CV events [15].

### Underlying cardiovascular comorbidities

Similarly to influenza virus infections [16, 17], people affected by SARS-CoV-2 with pre-existing CVD have an increased risk of developing acute myocardial injury [18], resulting in a poor prognosis. It has been described a more severe and acute systemic response to the infection in these patients, with increased leukocyte count, higher levels of cardiac necrosis biomarkers and greater incidence of ARDS [18]. In particular, patients with underlying CVD and higher levels of TnT showed the worst outcome (mortality 69.44%), as compared to both individuals with increased TnT but without CVD comorbidities (mortality 37.5%) and subjects with previous CVD and normal TnT (mortality 13.3%) [4]. As reported in previous studies, the overall percentage of pre-existing CVD in patients with COVID-19 was 24–48%, and the most frequent comorbidities were hypertension (15–31%), diabetes (7–20%), coronary artery disease (CAD) (3–8%) and other CVD (15%) [19–22]. The prevalence of these diseases was greater among patients admitted to the intensive care unit (ICU) [10].

As explained before, the genesis of CV involvement in COVID-19 remains debated. For what concerns cardiac injury in patients with CVD, several studies speculated about different etiologies of secondary cardiac involvement. The increase in myocardial oxygen demand during the infection could bring to a cardiac decompensation in patients with pre-existing heart failure [18], while the systemic inflammatory release of cytokines could lead to a major risk for atherosclerotic plaque rupture [21]. Then, the increased coagulation activity, expressed by increased D-dimer levels, could induce thrombosis and ischemia [21]. Furthermore, other studies hypothesized that hypoxia could reduce the oxygen supply to the heart, unmasking a CAD or a microvascular pathology causing myocardial infarction with non-obstructive coronary artery (MINOCA) [23].

Finally, in predisposed patients, hypoxia, together with drugs and systemic inflammation, could lead to arrhythmias.

## Myocarditis

Myocarditis is a well-recognized complication of acute viral infections [24], as a wide spectrum of viral genomes has been identified in the endomyocardial specimens of patients with clinical suspicion of myocarditis and parvovirus B-19, adenovirus or influenza infection [25].

Myocarditis has also been reported as a complication of middle east respiratory syndrome (MERS), caused by another severe coronavirus [26].

Several case reports of COVID-19 patients with acute myocardial injury, defined as troponin release, provide evidence of cardiac inflammation [27] with cardiac magnetic resonance (CMR) findings compatible with acute myocarditis [28, 29].

Notably, Esposito et al. [30] reported a series of 8 patients with elevated concentrations of TnT and electrocardiography alterations whom CMR findings fulfilled the 2018 Lake Louise Criteria for the diagnosis of myocarditis. In all cases, CMR showed diffuse intense myocardial edema, increased T1 and T2 mapping and also a mild pericardial effusion in 75% of patients.

Worth noticing, all patients had no remarkable previous history of cardiovascular disease.

Even Inciardi et al. [31] reported a case of a 53-year-old woman with no CV history presented to the emergency department with severe fatigue and abnormal ECG findings with elevated levels of markers of myocyte necrosis. After urgent invasive coronary angiography (ICA) was performed with no evidence of obstructive coronary disease, CMR showed marked biventricular myocardial interstitial edema and diffuse transmural late gadolinium enhancement (LGE) with circumferential pericardial effusion, with the final diagnosis of myopericarditis.

Although the gold standard in the diagnosis of myocarditis is endomyocardial biopsy (EMB), through histological, immunological and molecular evidences [32], it is rarely performed in COVID-19 patients for obvious implications related to the complexity of organizing a procedure which is invasive and poses all involved operators at risk of infection. In this setting, CMR represents the ideal noninvasive imaging tool for clinical diagnosis of myocarditis.

CMR patterns have been reported to be heterogeneous, as aforementioned, but in general not different from any other typical form of active inflammation characterized by diffuse myocardial edema. LGE seems to be less-frequently observed in these patients [30], reflecting a limited myocyte necrosis at least in acute phase, and highlighting the key role of the new Lake Louise Criteria in myocarditis diagnosis [33].

We noted similar findings in our experience with a crucial role of mapping techniques for the assessment of myocardial inflammation.

**Fig. 2:**
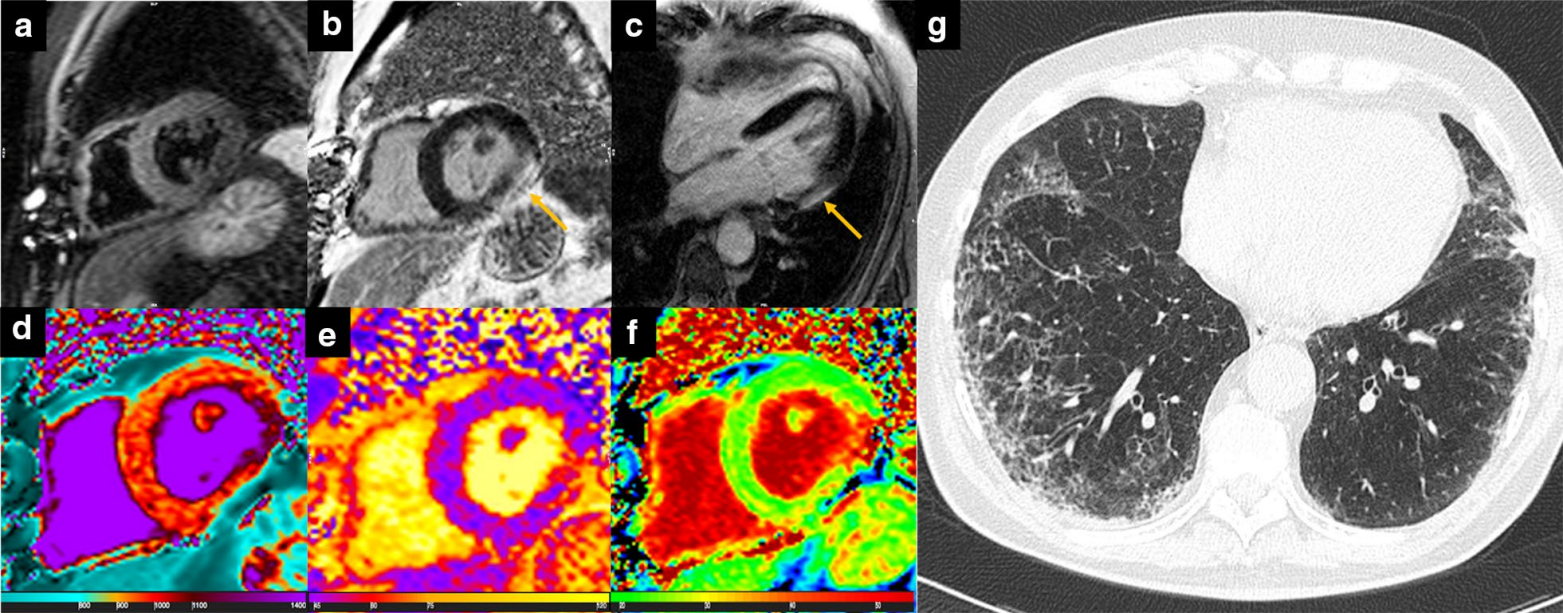
Myocarditis. 51-year-old man hospitalized for COVID‐19 pneumonia and sudden onset of tachyarrhythmias. STIR images revealed no edema (**a**), although LGE was evident on infero-lateral segments of basal-mid planes with a subepicardial pattern of distribution (**b**, **c** orange arrows) and native T1 was increased on LGE + segments (**d**). T2 mapping sequences revealed the presence of edema on infero-lateral segments of mid-ventricular planes (**e**). ECV confirmed those findings with implemented values on infero-lateral wall (**f**). Chest CT showed GGO predominantly distributed on inferior lobes with a peripheral distribution (**g**). *STIR* short tau inversion recovery, *LGE* late gadolinium enhancement, *ECV* extracellular volume, *CT* computed tomography, *GGO* ground glass opacity

When present LGE has a non-ischemic pattern, as in case showed in Fig. 2, with a predominant location at inferior and inferior-lateral segments [34].

In their echocardiographic study, Moody et al. have found an independent association between reduced RV systolic dysfunction and all-cause mortality in severe COVID-19 patients with elevated TnT. Besides the effects of thromboembolic disease, authors hypothesized a possible primary RV involvement as a concurrent factor to RV injury [35]. In this clinical setting, a significant contribution could be provided by CMR, which has shown to be the gold standard for the evaluation of RV function together with tissue characterization [36].

Up to date, histopathological confirmation of myocarditis was found in only one patient with CMR findings of reverse Takotsubo syndrome and final diagnosis of lymphocytic myocarditis, with no evidence of SARS-CoV-2 genome within the myocardium [37].

In a cohort study of 39 autopsy cases of patients with COVID-19, Lindner et al. found viral genome in the myocardial tissue in 61.5% of autopsies (*n* = 24/39), with virus load above 1000 copies in most cases (*n* = 16/24) [11].

Other autopsy studies [38, 39] showed inflammatory infiltrates in the heart tissue with no detection of viral genome, suggesting an indirect immune-mediated injury of the heart in COVID-19 patients.

In the largest CMR cohort study in patients recently recovered from COVID-19, Puntmann et al. found cardiac involvement in 78% of patients, detected by raised myocardial native T1 (*n* = 73), T2 (*n* = 60), LGE (*n* = 32) and pericardial enhancement (*n* = 22) [40].

Among these, 3 patients were referred to EMB with diagnosis of active lymphocytic inflammation, with no evidence of viral genome.

On the same line, another recent study demonstrated subclinical ongoing or resolving myocardial inflammation in athletes with previous COVID-19 infection [6]. In particular, CMR diagnosis of myocarditis was achieved in 15% (*n* = 4/26), while 30.8% additional athletes (*n* = 8) exhibited LGE without T2 elevation.

**Fig. 3:**
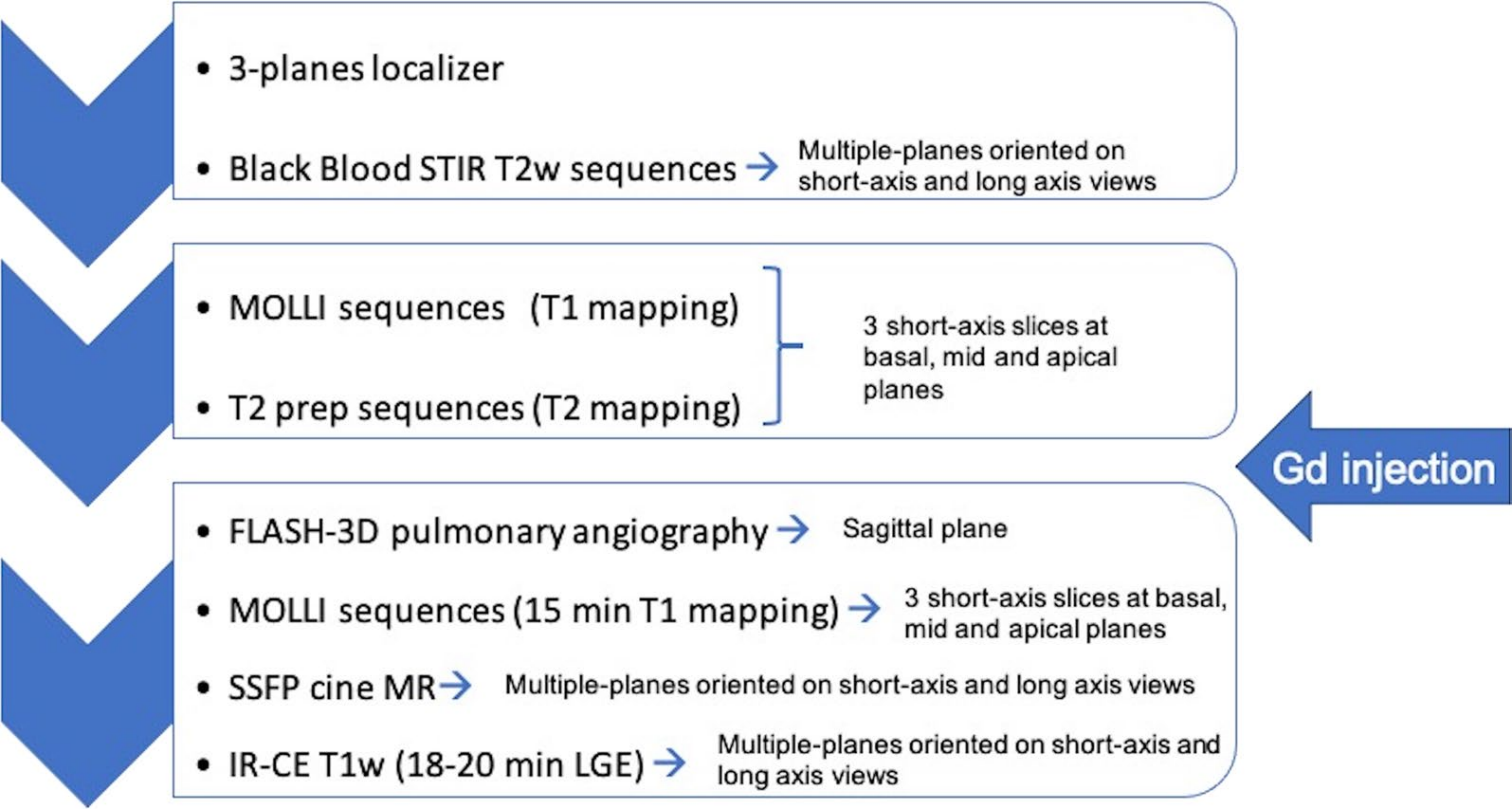
Suggested CMR protocol for myocarditis diagnosis in COVID-19 patients. *STIR* short tau inversion recovery; *MOLLI* modified look-locker imaging; *FLASH-3D* fast low angle shot three-dimensional; *SSFP* steady-state free precession; *MR* magnetic resonance; *IR-CE* inversion recovery contrast enhanced; *LGE* late gadolinium enhancement; *Gd* gadolinium

Although the underlying mechanism remains unclear, whether the cardiac injury is directly induced by viral replication in the myocardium or conveyed by an exaggerated immune system response, myocarditis-like syndrome seems to be one of the main phenotypical expression of cardiac injury in COVID-19 patients. In this scenario CMR has a pivotal role in diagnosis and patients’ risk stratification. Our proposed CMR protocol is summarized in Fig. 3.

## Pericarditis

Association of SARS-CoV-2 infection and acute pericarditis is currently poorly documented.

To the best of our knowledge, no studies investigating the prevalence of this manifestation have been published yet, despite the non-infrequent detection of effusion in bedside echocardiography observed in our clinical practice.

Nevertheless, viral infection is the leading cause of pericarditis in developed countries [41] and the virus has been isolated in pericardial fluid of a patients with cardiac tamponade [42].

In the past months, few papers described cases of pericardial involvement in COVID-19 patients, either in association with myocarditis or as isolated manifestation [8, 43–46].

Although pericarditis is not a common complication in COVID-19, it should be promptly considered in patients with chest pain, ST elevation on ECG and normal coronary angiogram; a misdiagnosis, indeed, can produce life-threatening consequences, such as cardiac tamponade [47].

**Fig. 4:**
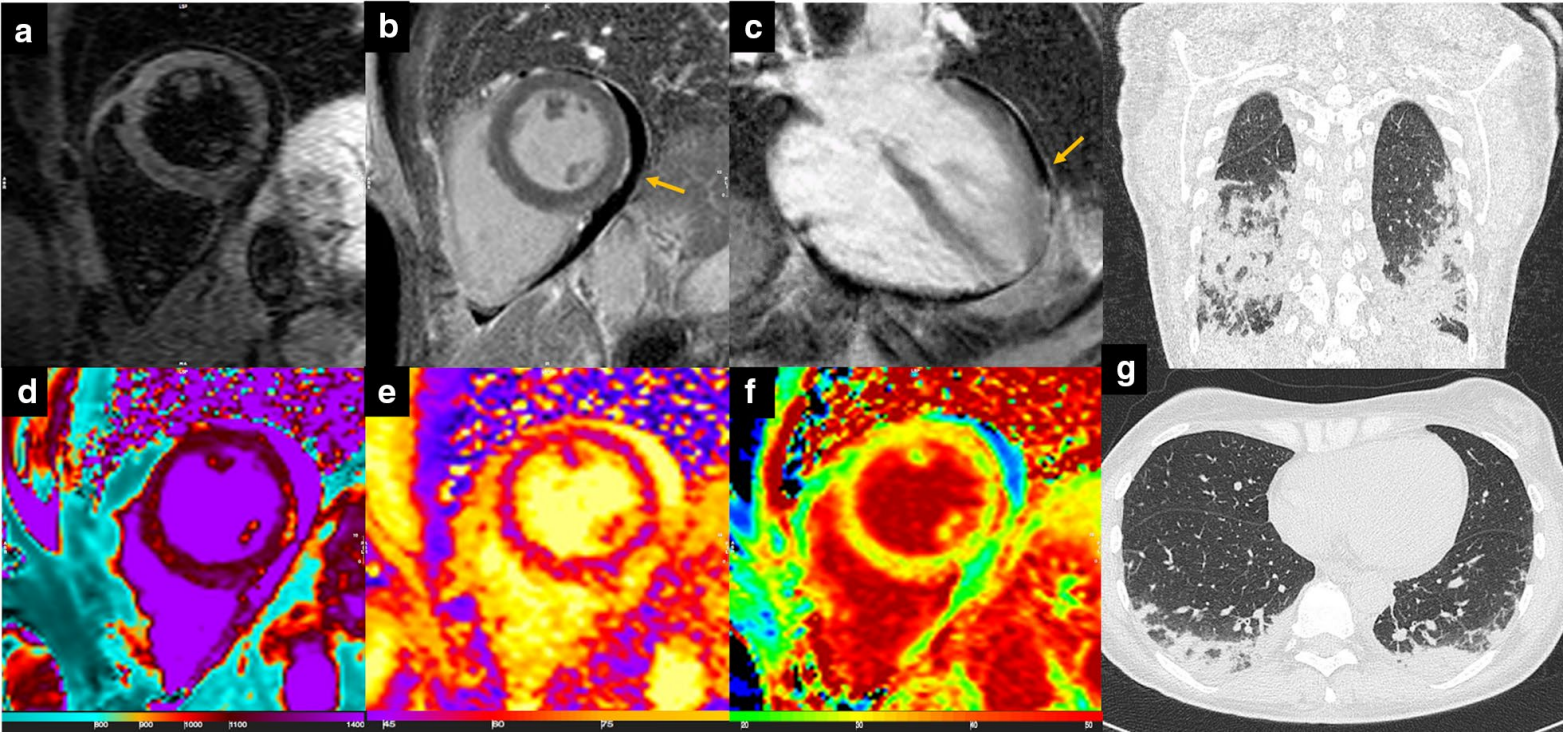
Pericarditis. 36-year-old in-hospital COVID-19 patient with chest discomfort and evidence of pericardial effusion at bedside Echo. **a** Evidence a slight hyperintensity of the pericardial layers, with no myocardial edema. LGE sequences confirm the presence on pericardial layers enhancement and moderate pericardial effusion (**b**, **c** orange arrows). Native T1 values were mildly increased (**d**) as well as T2 mapping (**e**) and ECV values (**f**). Coronal and axial chest CT revealed peripheral posterior areas of parenchymal consolidation (**g**). *Echo* echocardiography; *STIR* short tau inversion recovery; *LGE* late gadolinium enhancement; *ECV* extracellular volume; *CT* computed tomography

Despite the fact that computed tomography (CT) and CMR are not included in diagnostic criteria for pericarditis [48], these imaging modalities can provide supportive findings and are strongly recommended as second-level testing for diagnostic workup of the pathology [49]. CMR hallmark in acute pericarditis includes the combination of diffusely edematous and enhancing pericardial layers with a variable amount of effusion, as shown in Fig. 4. This may serve not only to confirm the diagnosis, but also to rule-out alternative overlapped diagnoses such as myocarditis or pulmonary embolism.

Additionally, in COVID-19 era, conventional echocardiography (i.e. not bedside) is potentially underused due to an increased infection risk for healthcare providers during the examination [50].

## Myocardial Infarction: obstructive versus MINOCA syndromes

As mentioned above, SARS-CoV-2 infection can increase the risk of ACS [51]. This finding was confirmed by previous studies conducted on influenza virus [17] and community acquired pneumonia [52], demonstrating an improved risk of acute myocardial infarction (AMI), which was maximum within 7 days from the diagnosis in the affected patients [53].

Uncontrolled inflammatory response has been observed to be the most important determinant of ACS in COVID-19: stimulation of macrophages, endothelial and smooth muscle cells, activation of platelets and the expression of tissue factor in the atheromatous plaque concur to plaque rupture and ACS [24]. The effective incidence of ACS among patients with COVID-19 is still unknown and probably underestimated, due to the relative inaccessibility to healthcare services during the pandemic [10, 32]. For the same reasons, we do not have a realistic ACS risk evaluation in COVID-19 patients without CVD.

According to the American College of Cardiology, patients with diagnosis of ST-Elevation Myocardial Infarction (STEMI) should undergo ICA, for a combined diagnostic and therapeutic approach.

As interesting remark, a delay in revascularization of STEMI patients has been reported in Italy during the first wave of COVID-19 outbreak [50], highlighting the logistical difficulties in the management of even the most critical medical conditions during the pandemic.

For an imaging perspective, this could lead to the observation of subacute patterns, with mild, if not reabsorbed, myocardial edema and initial healing of myocyte necrosis.

For patients with active SARS-CoV-2 infection and Non-ST Elevation Myocardial Infarction (NSTEMI), diagnostic testing prior to catheterization is recommended [51]. In hemodynamically stable cases and equivocal presentation, cardiac CT could be considered as a valid alternative to ICA [54]. CT allows the comprehensive assessment of pulmonary parenchyma, pulmonary vessels and coronary arteries and allows to rule-out CAD with a nearly100% negative predictive value [23].

Pontone et al. reported their experience performing cardiac CT in a patient with dyspnea and chest pain tested positive for SARS-CoV-2, proposing a CT protocol to “quadruple rule-out” out pneumonia, pulmonary embolism, myocarditis and ACS in selected COVID-19 patients [54].

**Fig. 5:**
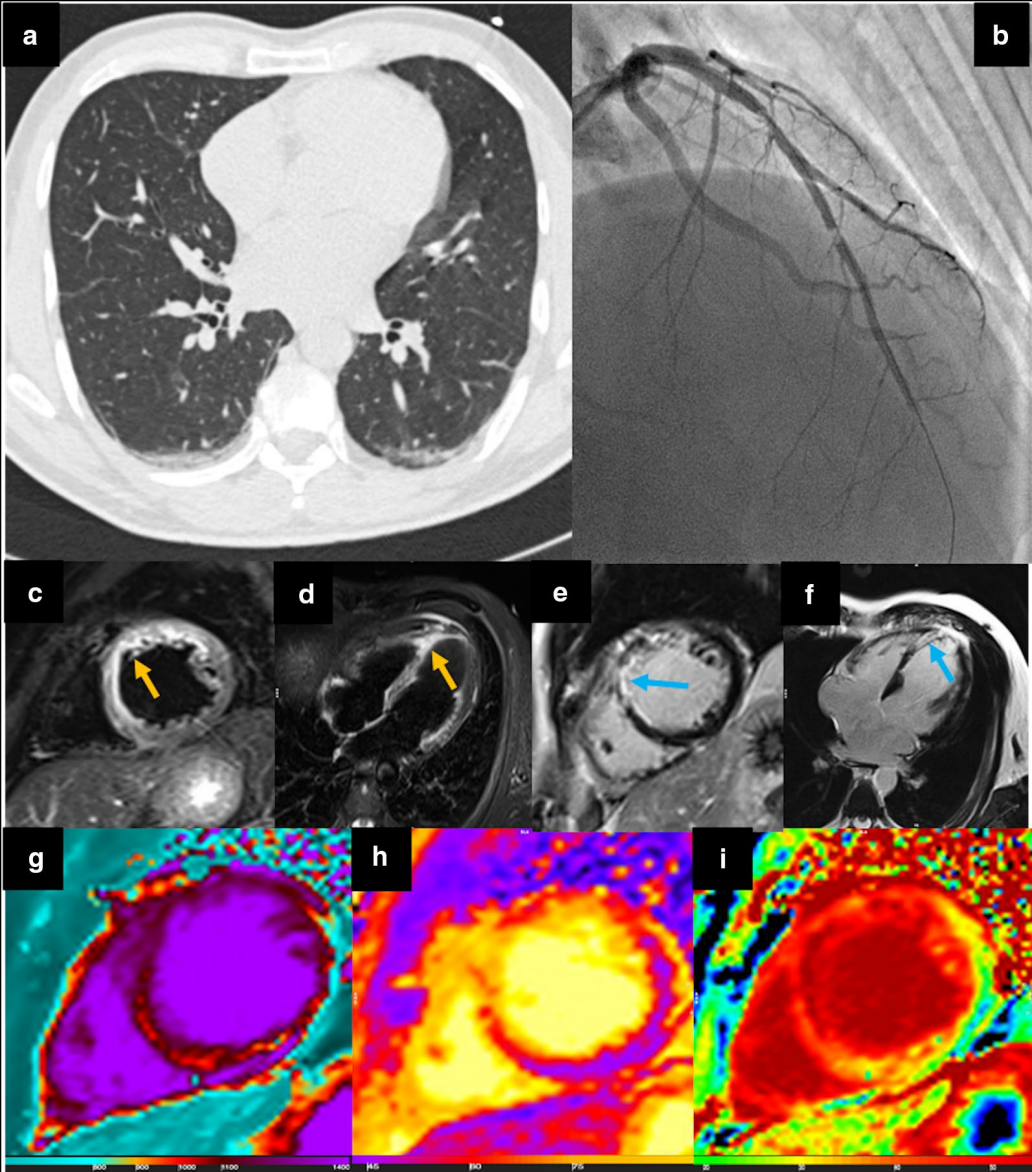
STEMI. 45-year-old male patient with no CV history admitted to ER with acute chest pain, elevation of TnT and ST elevation at ECG. Nasopharyngeal swab tested positive for SARS-CoV-2 and chest CT showed a mild pulmonary involvement (**a**). Occlusion of proximal DA was detected and treated at ICA (**b**). CMR, performed 7 days after revascularization, showed antero-septal edema of mid and apical planes at STIR images (**c**, **d** orange arrows). LGE sequences evidenced necrosis with subendocardial distribution on antero-septal segments and transmural extension on anterior segments on mid planes (**e**, **f** blue arrows). Increased values of native T1 (**g**), T2 mapping (**h**) and ECV fraction (**i**) confirmed the presence of edema and necrosis on anterior and antero-septal segments on mid and apical planes. *CV* cardiovascular; *ER* emergency room; *TnT* t-troponin; *CT* computed tomography; *DA* descending coronary artery; *ICA* invasive coronary angiography; *CMR* cardiac magnetic resonance; *STIR* short tau inversion recovery; *LGE* late gadolinium enhancement, *ECV* extracellular volume

CMR findings of AMI include myocardial edema, detected by T2-weighted short tau inversion recovery (STIR) and confirmed by elevated T2 mapping values, along with matched subendocardial distribution of LGE, as shown in Fig. 5.

After excluding CAD, other possible differential diagnoses of AMI should be investigated. [23].

Several articles reported the evidence of MINOCA or AMI type 2 among patients with COVID-19. The former explained with the presence of high levels of ACE-2 receptors in pericytes and in endothelial cells, which hampers a severe microvascular dysfunction also associated with cytokines storm [24]. The latter caused by a mismatch between oxygen supply and demand [55]. In both of these clinical entities, ICA would be of limited use [24] and other imaging technique should be considered in order to exclude other CVD with similar clinical presentations [23], such as myocarditis with infarct-like onset and Takotsubo cardiomyopathy.

Accordingly, few cases of Takotsubo syndrome have been reported, likely triggered by both intense emotional stress and systemic inflammation COVID-19 related [37, 56, 57].

**Fig. 6:**
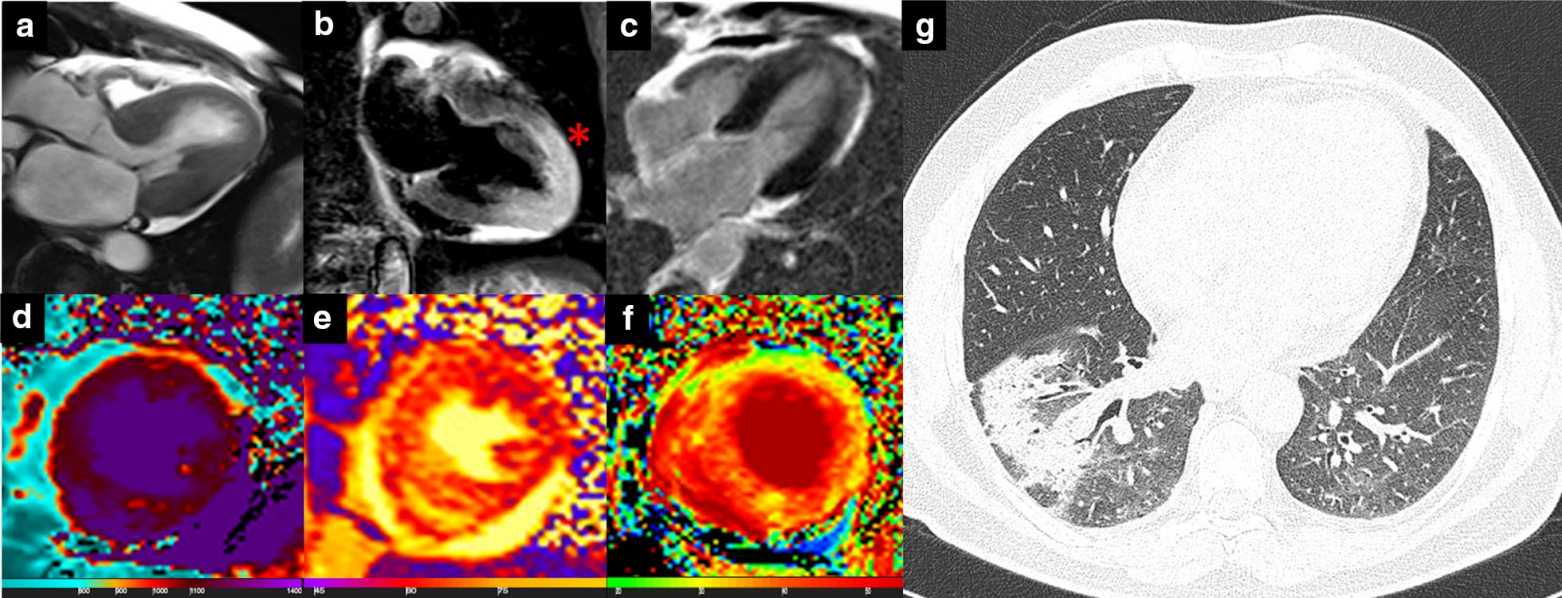
Takotsubo cardiomyopathy. COVID-19 female patient with chest pain and no CAD. Cine-SSFP showed a diffuse hypokinesia of mid and apical segments with «apical ballooning» sign (**a**). Diffuse edema of mid-ventricular and apical segments on 2-chambers STIR was found (**b** red asterisk). PSIR sequences evidenced no LGE (**c)**. Native T1, T2 mapping and ECV fraction values were all increased (**d**–**f**). Chest CT showed a low-grade pulmonary involvement with peripheral interstitial thickening (**g**). *CAD* coronary artery disease; *SSFP* steady-state free precession; *STIR* short tau inversion recovery; *PSIR* phase sensitive inversion recovery; *LGE* late gadolinium enhancement; *ECV* extracellular volume; *CT* computed tomography

CMR findings of Takotsubo syndrome in COVID-19 patients showed no significant differences from the classic pattern: a diffuse hyperintensity in T2 STIR images in mid-apical segments matched the area of regional dysfunction [58] (Fig. 6).

CMR has been poorly used to investigate myocardial injury in COVID-19 patients so far, especially during the acute phases of the disease [28, 30, 40, 59], and MINOCA phenotypes could reasonably be underdiagnosed in this group.

In selected COVID-19 patients, CMR allows to noninvasively characterize underlying pathological substrate, offering a comprehensive evaluation of biventricular morphology, function and myocardial tissue characterization.

Further studies are needed to better define the role of CMR in this subset of patients.

## Arrhythmias and sudden cardiac arrest: role of advanced imaging?

Cardiac arrhythmias, of both atrial and ventricular origin, are a common complication in SARS-CoV-2 infection [60, 61].

In the report by Colon et al., 19 out of 119 (16.5%) patients, all admitted in ICU, developed new-onset atrial arrhythmias, including atrial fibrillation (63%), atrial flutter (32%) and atrial tachycardia (5%) [62]. Atrioventricular/ventricular block and QT prolongation have been reported in association with SARS-CoV-2 infection, with incidence of 11.8% and 13% respectively [60]. Malignant arrhythmias, as ventricular tachycardia and fibrillation, have been described as well since early reports from China, especially in patients with elevated TnT levels [4].

Furthermore, sudden cardiac arrest was reported as fatal outcome in COVID-19 patients, in both in—[63] and out-of-hospital settings [64].

A special mention is finally needed for arrhythmogenic effects of COVID-19-related drugs. Specifically, antiviral therapies, such as Lopinavir and Ritonavir, are associated with atrioventricular block while azithromycin, chloroquine and hydroxychloroquine with QT prolongation [10]. Although there are no indications in the acute setting, advanced cardiac imaging is an essential tool in the assessment of dysrhythmias, improving sudden cardiac death risk stratification. CMR provides reliable and reproducible evaluation of structural changes and can be applied to identify underlying arrhythmogenic substrate (e.g. myocardial edema, ischemia and/or fibrosis). Thus, it could play a role to identify pathological substrates in patients with new onset arrhythmias and to improve arrhythmic event prediction in selected patients recovered from COVID-19 with infection-related cardiac injury.

## Coagulation abnormalities and pulmonary embolism

Thromboembolic disease represents another piece in the mosaic of SARS-CoV-2 infection, which is commonly observed in COVID-19 and associated with poor prognosis. Increased D-dimer levels in COVID-19 patients have been early reported in China [65], with higher levels in patients in ICU [median IQR 414 (191–1324) vs. 166 (101–285), *p* value < 0.001] [20].

Fei Zhou et al., in one of the largest retrospective cohort studies, not only reported increased coagulation activity in about 90% of COVID-19 in patients affected by pneumonia, but also pointed out elevated D-dimer levels at admission to be one of the main risk factors of in-hospital death [21].

It has not been fully elucidated yet whether this hypercoagulable state depends on a direct endothelial damage inflicted by the virus or represents a consequence of cytokine storm precipitating the onset of SIRS [66]. The blood coagulation activation in COVID-19, also substantiated by elevated levels of fibrinogen, Von Willebrand factor activity and factor VIII, seems not meet the criteria for disseminate intravascular coagulation, as platelet count and prothrombin time have been mostly reported within normal ranges, or just slightly increased, in these patients [67, 68].

Moreover, Helms et al. reported in ICU population with ARDS a much higher incidence of pulmonary embolism (PE) in COVID-19 patients than in non-COVID-19 (11.7 vs. 2.1%) [67].

Reasonably, SARS-CoV-2-associated thromboembolic disease has a multifactorial etiology, clinically revealed mainly by the onset of PE.

**Fig. 7:**
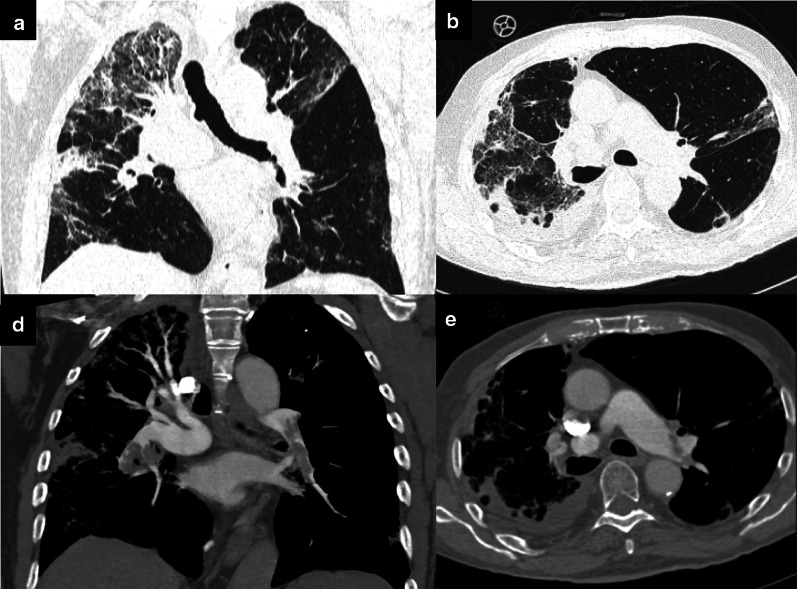
Pulmonary Embolism. COVID-19 patient with dyspnea, increased D-dimer levels and O2 saturation < 93. Chest CT evidenced GGO with interstitial thickening of the interlobular and intralobular septa, together with pleural effusion (**a**, **b**). After contrast media injection, PE was found in the left upper lobar branch, the left inferior lobar branch and the segmental arteries for the left inferior pulmonary lobe and PE of the interlobar arteries for the inferior right lobe, probably extended to the subsegmentary arteries (**d**, **e**). *CT* computed tomography; *GGO* ground glass opacity; *PE* pulmonary embolism

In a retrospective study conducted in France among 100 COVID-19 inpatients undergone CT pulmonary angiography (CTPA), 23% had acute PE. This group was more frequently in ICU (17 [74%] vs. 22 [29%]; *P*, 0.001) than non-PE-group and required mechanical ventilation more often (15 of 23 patients [65%] vs. 19 of 77 patients [25%], *P*, 0.001) [69]. Several other studies with CTPA confirmed these findings [67, 70, 71], raising the needing of a more defined role of contrast chest CT in COVID-19 diagnostic work-up. CTPA is the imaging modality of choice in clinical suspicion of PE [72], and although is still not recommended in COVID-19 patients by current guidelines [73–75] it could significantly impact clinical outcome in selected groups, such as patients with elevated D-dimer levels, sudden clinical worsening and/or new onset of dyspnea (Fig. 7).

## Recommended patient’s workflow in suspected COVID-19

**Fig. 8:**
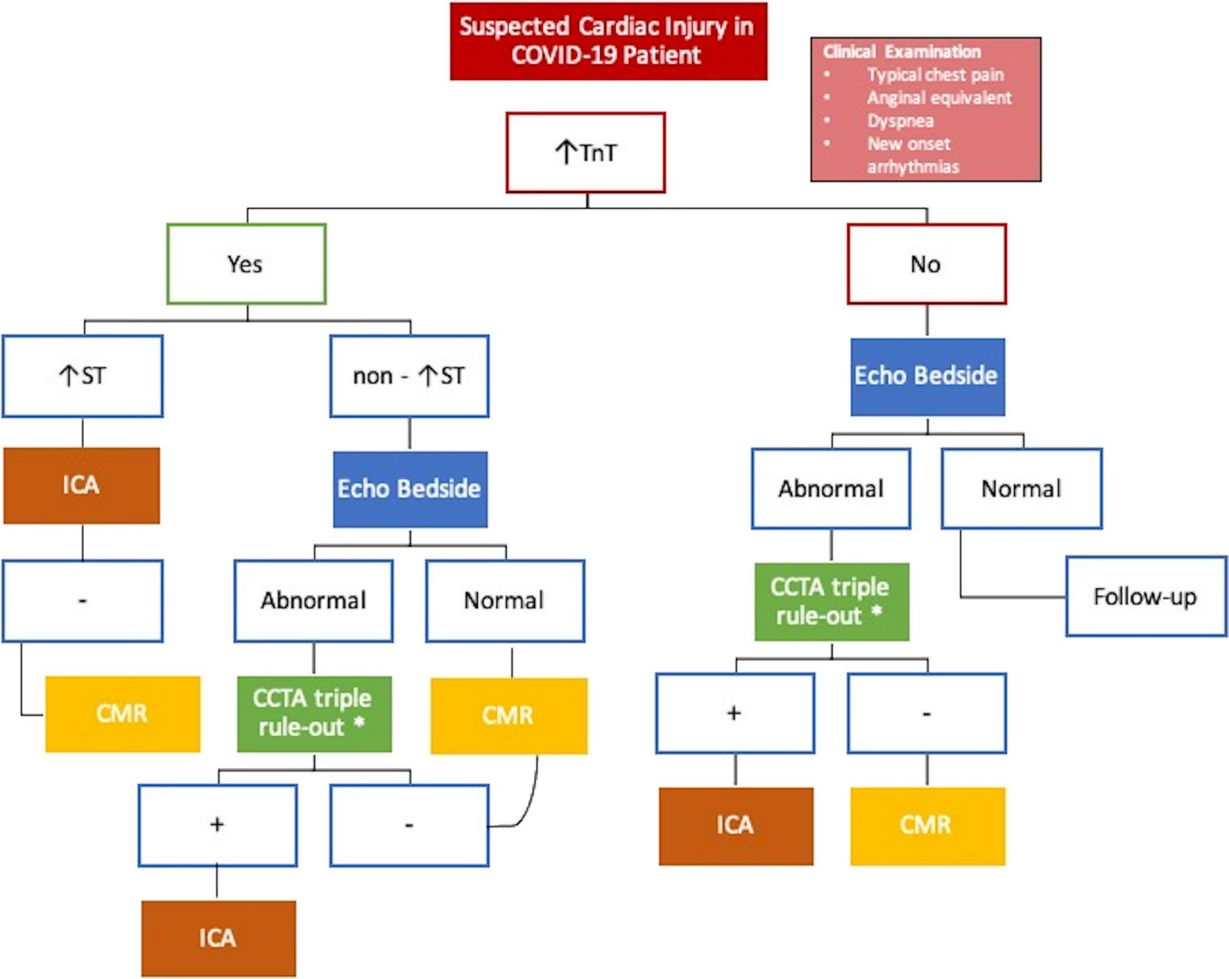
Suggested flowchart for imaging management of suspected cardiac injury in COVID-19 patients. (*) CCTA can be considered to triple rule-out CAD, interstitial pneumonia and pulmonary embolism in selected patients. *TnT* T-troponin; *Echo* echocardiography; *ICA* invasive coronary angiography; *CCTA* cardiac computed tomography angiography; *CMR* cardiac magnetic resonance

In clinical suspicion of CV involvement in COVID-19 patients, supported by typical or atypical chest pain, worsening of dyspnea and/or new onset of arrhythmias, bedside echocardiography represents the first essential imaging tool in diagnostic work-up. Considering the high exposure risk for operators, it is not routinely performed in COVID-19 patients. Anyway, a fast and focused bedside echocardiography evaluation can be pivotal in referring selected patients to second level imaging, such as cardiac CT, CMR and ICA, to avoid unnecessary examinations and diagnostic delay, as shown in Fig. 8.

According to our proposed algorithm, cardiac CT can be used to rule-out ACS when coronary obstruction is suspected, while CMR plays a key role in the differential diagnosis of myocardial injury with normal coronary arteries [76].

Safety precautions should be implemented to prevent risk of infection for both healthcare professionals and patients. If available, utilization of COVID-19 dedicated scanners with appropriate cleaning and decontamination of the area at the end of the shift would be highly recommended.

A full personal protective equipment is needed for healthcare staff, which need to be reduced to the minimum necessary, while all patients should wear surgical masks [77, 78].

In our experience, we implemented the use of rapid and tailored CT and CMR protocols to reduce exposure time related infection risk and patient discomfort [79].

## Conclusions and future perspectives

Rationale utilization of advanced cardiac imaging services in COVID-19 should serve to avoid or, at least, minimize the unnecessary use of invasive, potentially contagious and time-consuming procedures like cardiac catheterization or transesophageal echocardiography and to speed-up diagnostic pathways.

While bedside echocardiography remains a first-line and ease-of-use diagnostic tool, appropriate use of second-line techniques, such as cardiac CT and CMR, allows the reliable exclusion of CAD together with the characterization of underlying pathological substrate (see Fig. 8).

Our clinical experience, also supported by scientific literature [40], has shown that a progressive shift from acute to chronic cardiovascular damage is not uncommon, even in apparently healed patients and includes conditions like chronic thrombo-embolic pulmonary hypertension, recurrent arrhythmias and progression to dilated cardiomyopathy. Accordingly, careful follow-up of these patients should be offered in the convalescent stage.

In conclusion, besides pulmonary manifestation, radiologists should be aware of the wide and heterogeneous spectrum of cardiovascular complications in COVID-19 and rationalized utilization of advanced imaging tools which may be used to drive therapeutic approach and stratify patients’ prognosis.

## Data Availability

Not applicable.

## Abbreviations

ACE2: Angiotensin-converting enzyme 2
ACS: Acute coronary syndrome
AMI: Acute myocardial infarction
ARDS: Acute respiratory distress syndrome
CAD: Coronary artery disease
CMR: Cardiovascular magnetic resonance
COVID-19: Coronavirus disease 2019
CT: Computed tomography
CTPA: Computed tomography pulmonary angiography
CV: Cardiovascular
CVD: Cardiovascular disease
DWI: Diffusion-weighted imaging
ECV: Extra-cellular volume
ICA: Invasive coronary angiography
ICU: Intensive care unit
LGE: Late gadolinium enhancement
MERS: Middle east respiratory syndrome
MINOCA: Myocardial infarction with non-obstructive coronary artery
MOF: Multiorgan failure
NSTEMI: Non-ST elevation myocardial infarction
PE: Pulmonary embolism
ROS: Reactive oxygen species
SARS-CoV-2: Severe acute respiratory syndrome coronavirus 2
SIRS: Systemic inflammatory response syndrome
STEMI: ST-elevation myocardial infarction
STIR: Short Tau inversion recovery
TnT: T-troponin

## References

[1] Guzik TJ, Mohiddin SA, Dimarco A (2020). COVID-19 and the cardiovascular system: implications for risk assessment, diagnosis, and treatment options. Cardiovasc Res.

[2] Zheng YY, Ma YT, Zhang JY, Xie X (2020). COVID-19 and the cardiovascular system. Nat Rev Cardiol.

[3] Shi S, Qin M, Shen B (2020). Association of cardiac injury with mortality in hospitalized patients with COVID-19 in Wuhan, China. JAMA Cardiol.

[4] Guo T, Fan Y, Chen M (2020). Cardiovascular implications of fatal outcomes of patients with coronavirus disease 2019 (COVID-19). JAMA Cardiol.

[5] Luetkens JA, Isaak A, Zimmer S (2020). Diffuse myocardial inflammation in COVID-19 associated myocarditis detected by multiparametric cardiac magnetic resonance imaging. Circ Cardiovasc Imaging.

[6] Rajpal S, Tong MS, Borchers J (2020). Cardiovascular magnetic resonance findings in competitive athletes recovering from COVID-19 infection. JAMA Cardiol.

[7] Bangalore S, Sharma A, Slotwiner A (2020). ST-segment elevation in patients with Covid-19—a case series. N Engl J Med.

[8] Sauer F, Dagrenat C, Couppie P (2020). Pericardial effusion in patients with COVID-19: case series. Eur Hear J Case Rep.

[9] Wu L, O’Kane AM, Peng H (2020). SARS-CoV-2 and cardiovascular complications: from molecular mechanisms to pharmaceutical management. Biochem Pharmacol.

[10] Nishiga M, Wang DW, Han Y (2020). COVID-19 and cardiovascular disease: from basic mechanisms to clinical perspectives. Nat Rev Cardiol.

[11] Lindner D, Fitzek A, Bräuninger H (2020). Association of cardiac infection with SARS-CoV-2 in confirmed COVID-19 autopsy cases. JAMA Cardiol.

[12] Oudit GY, Kassiri Z, Jiang C (2009). SARS-coronavirus modulation of myocardial ACE2 expression and inflammation in patients with SARS. Eur J Clin Invest.

[13] Ferrario CM, Chappell MC, Tallant EA (1997). Counterregulatory actions of angiotensin-(1-7). Hypertension.

[14] Li B, Yang J, Zhao F (2020). Prevalence and impact of cardiovascular metabolic diseases on COVID-19 in China. Clin Res Cardiol.

[15] Corrales-Medina VF, Musher DM, Shachkina S, Chirinos JA (2013). Acute pneumonia and the cardiovascular system. Lancet.

[16] Nguyen JL, Yang W, Ito K (2016). Seasonal influenza infections and cardiovascular disease mortality. JAMA Cardiol.

[17] Kwong JC, Schwartz KL, Campitelli MA (2018). Acute myocardial infarction after laboratory-confirmed influenza infection. N Engl J Med.

[18] Bonow RO, O’Gara PT, Yancy CW (2020). Cardiology and COVID-19. JAMA J Am Med Assoc.

[19] Huang C, Wang Y, Li X (2020). Clinical features of patients infected with 2019 novel coronavirus in Wuhan, China. Lancet.

[20] Wang D, Hu B, Hu C (2020). Clinical characteristics of 138 hospitalized patients with 2019 novel coronavirus-infected pneumonia in Wuhan, China. JAMA J Am Med Assoc.

[21] Zhou F, Yu T, Du R (2020). Clinical course and risk factors for mortality of adult inpatients with COVID-19 in Wuhan, China: a retrospective cohort study. Lancet.

[22] Guan W, Ni Z, Hu Y (2020). Clinical characteristics of coronavirus disease 2019 in China. N Engl J Med.

[23] Agricola E, Beneduce A, Esposito A (2020). Heart and lung multimodality imaging in COVID-19. JACC Cardiovasc Imaging.

[24] Guzik TJ, Mohiddin SA, Dimarco A (2020). COVID-19 and the cardiovascular system: implications for risk assessment, diagnosis, and treatment options. Cardiovasc Res.

[25] Fung G, Luo H, Qiu Y (2016). Myocarditis. Circ Res.

[26] Alhogbani T (2016). Acute myocarditis associated with novel Middle East respiratory syndrome coronavirus. Ann Saudi Med.

[27] Zeng JH, Liu YX, Yuan J (2020). First case of COVID-19 complicated with fulminant myocarditis: a case report and insights. Infection.

[28] Kim IC, Kim JY, Kim HA, Han S (2020). COVID-19-related myocarditis in a 21-year-old female patient. Eur Heart J.

[29] Trogen B, Gonzalez FJ, Shust GF (2020). COVID-19-associated myocarditis in an adolescent. Pediatr Infect Dis J.

[30] Esposito A, Palmisano A, Natale L (2020). Cardiac magnetic resonance characterization of myocarditis-like acute cardiac syndrome in COVID-19. JACC Cardiovasc Imaging.

[31] Inciardi RM, Lupi L, Zaccone G (2020). Cardiac involvement in a patient with coronavirus disease 2019 (COVID-19). JAMA Cardiol.

[32] Peretto G, Sala S, Caforio ALP (2020). Acute myocardial injury, MINOCA, or myocarditis? Improving characterization of coronavirus-associated myocardial involvement. Eur Heart J.

[33] Cundari G, Galea N, De Rubeis G (2020). Use of the new Lake Louise Criteria improves CMR detection of atypical forms of acute myocarditis. Int J Cardiovasc Imaging.

[34] Huang L, Zhao P, Tang D (2020). Cardiac involvement in patients recovered from COVID-2019 identified using magnetic resonance imaging. JACC Cardiovasc Imaging.

[35] Moody WE, Mahmoud-Elsayed HM, Senior J (2020). Impact of right ventricular dysfunction on mortality in patients hospitalized with COVID-19, according to race. CJC Open.

[36] Galea N, Carbone I, Cannata D (2013). Right ventricular cardiovascular magnetic resonance imaging: normal anatomy and spectrum of pathological findings. Insights Imaging.

[37] Sala S, Peretto G, Gramegna M (2020). Acute myocarditis presenting as a reverse Tako-Tsubo syndrome in a patient with SARS-CoV-2 respiratory infection. Eur Heart J.

[38] Tian S, Xiong Y, Liu H (2020). Pathological study of the 2019 novel coronavirus disease (COVID-19) through postmortem core biopsies. Mod Pathol.

[39] Xu Z, Shi L, Wang Y (2020). Pathological findings of COVID-19 associated with acute respiratory distress syndrome. Lancet Respir Med.

[40] Puntmann VO, Carerj ML, Wieters I (2020). Outcomes of cardiovascular magnetic resonance imaging in patients recently recovered from coronavirus disease 2019 (COVID-19). JAMA Cardiol.

[41] Imazio M, Spodick DH, Brucato A (2010). Diagnostic issues in the clinical management of pericarditis. Int J Clin Pract.

[42] Farina A, Uccello G, Spreafico M (2020). SARS-CoV-2 detection in the pericardial fluid of a patient with cardiac tamponade. Eur J Intern Med.

[43] Blagojevic NR, Bosnjakovic D, Vukomanovic V (2020). Acute pericarditis and SARS-CoV-2: case report. Int J Infect Dis.

[44] Dabbagh MF, Aurora L, D’Souza P (2020). Cardiac tamponade secondary to COVID-19. JACC Case Rep.

[45] Purohit R, Kanwal A, Pandit A (2020). Acute myopericarditis with pericardial effusion and cardiac tamponade in a patient with COVID-19. Am J Case Rep.

[46] Asif T, Kassab K, Iskander F, Alyousef T (2020). Acute pericarditis and cardiac tamponade in a patient with COVID-19: a therapeutic challenge. Eur J Case Reports Intern Med.

[47] Hua A, O’Gallagher K, Sado D, Byrne J (2020). Life-threatening cardiac tamponade complicating myo-pericarditis in COVID-19. Eur Heart J.

[48] Maisch B, Seferović PM, Ristić AD (2004). Guidelines on the diagnosis and management of pericardial diseases: executive summary. The task force on the diagnosis and management of pericardial diseases of the European Society of Cardiology. Eur Heart J.

[49] Adler Y, Charron P, Imazio M (2015). 2015 ESC guidelines for the diagnosis and management of pericardial diseases. Eur. Heart J.

[50] Kirkpatrick JN, Mitchell C, Taub C (2020). ASE statement on protection of patients and echocardiography service providers during the 2019 novel coronavirus outbreak: endorsed by the American College of Cardiology. J Am Soc Echocardiogr.

[51] Long B, Brady WJ, Koyfman A, Gottlieb M (2020). Cardiovascular complications in COVID-19. Am J Emerg Med.

[52] Corrales-Medina VF, Alvarez KN, Weissfeld LA (2015). Association between hospitalization for pneumonia and subsequent risk of cardiovascular disease. JAMA J Am Med Assoc.

[53] Akhmerov A, Marbán E (2020). COVID-19 and the heart. Circ Res.

[54] Pontone G, Baggiano A, Conte E (2020). “Quadruple rule out” with cardiac computed tomography in COVID-19 patient with equivocal acute coronary syndrome presentation. JACC Cardiovasc Imaging.

[55] DeFilippis AP, Nasir K, Blaha MJ (2019). Myocardial infarction as a clinical end point in research. Circ Res.

[56] Meyer P, Degrauwe S, Van Delden C (2020). Typical takotsubo syndrome triggered by SARS-CoV-2 infection. Eur Heart J.

[57] Oyarzabal L, Gómez-Hospital JA, Comin-Colet J (2020). Síndrome de tako-tsubo asociado con COVID-19. Rev Española Cardiol.

[58] Iacucci I, Carbone I, Cannavale G (2013). Myocardial oedema as the sole marker of acute injury in Takotsubo cardiomyopathy: a cardiovascular magnetic resonance (CMR) study. Radiol Med.

[59] Ng M-Y, Ferreira VM, Leung ST (2020). Recovered COVID-19 patients show ongoing subclinical myocarditis as revealed by cardiac magnetic resonance imaging. JACC Cardiovasc Imaging.

[60] Wang Y, Wang Z, Tse G (2020). Cardiac arrhythmias in patients with COVID-19. J Arrhythmia.

[61] Liu K, Fang YY, Deng Y (2020). Clinical characteristics of novel coronavirus cases in tertiary hospitals in Hubei Province. Chin Med J (Engl).

[62] Colon CM, Barrios JG, Chiles JW (2020). Atrial arrhythmias in COVID-19 patients. JACC Clin Electrophysiol.

[63] Thapa SB, Kakar TS, Mayer C, Khanal D (2020). Clinical outcomes of in-hospital cardiac arrest in COVID-19. JAMA Intern Med.

[64] Baldi E, Sechi GM, Mare C (2020). Out-of-hospital cardiac arrest during the covid-19 outbreak in Italy. N Engl J Med.

[65] Chen N, Zhou M, Dong X (2020). Epidemiological and clinical characteristics of 99 cases of 2019 novel coronavirus pneumonia in Wuhan, China: a descriptive study. Lancet.

[66] Bikdeli B, Madhavan MV, Jimenez D (2020). COVID-19 and thrombotic or thromboembolic disease: implications for prevention, antithrombotic therapy, and follow-up: JACC state-of-the-art review. J Am Coll Cardiol.

[67] Helms J, Tacquard C, Severac F (2020). High risk of thrombosis in patients with severe SARS-CoV-2 infection: a multicenter prospective cohort study. Intensive Care Med.

[68] Panigada M, Bottino N, Tagliabue P (2020). Hypercoagulability of COVID-19 patients in intensive care unit: a report of thromboelastography findings and other parameters of hemostasis. J Thromb Haemost.

[69] Grillet F, Behr J, Calame P (2020). Acute pulmonary embolism associated with COVID-19 pneumonia detected by pulmonary CT angiography. Radiology.

[70] Leonard-Lorant I, Delabranche X, Severac F (2020). Acute pulmonary embolism in COVID-19 patients on CT angiography and relationship to D-dimer levels. Radiology.

[71] Poissy J, Goutay J, Caplan M (2020). Pulmonary embolism in patients with COVID-19: awareness of an increased prevalence. Circulation.

[72] Konstantinides SV, Meyer G, Bueno H (2020). 2019 ESC Guidelines for the diagnosis and management of acute pulmonary embolism developed in collaboration with the European Respiratory Society (ERS). Eur Heart J.

[73] Rubin GD, Haramati LB, Kanne JP (2020). The role of chest imaging in patient management during the COVID-19 pandemic: a multinational consensus statement from the Fleischner society. Radiology.

[74] Revel MP, Parkar AP, Prosch H (2020). COVID-19 patients and the radiology department—advice from the European Society of Radiology (ESR) and the European Society of Thoracic Imaging (ESTI). Eur Radiol.

[75] Simpson S, Kay FU, Abbara S (2020). Radiological society of north america expert consensus statement on reporting chest CT findings related to COVID-19. Endorsed by the Society of Thoracic Radiology, the American College of Radiology, and RSNA. Radiol Cardiothorac Imaging.

[76] Esposito A, Gallone G, Palmisano A (2020). The current landscape of imaging recommendations in cardiovascular clinical guidelines: toward an imaging-guided precision medicine. Radiol Med.

[77] Han Y, Chen T, Bryant J (2020). Society for Cardiovascular Magnetic Resonance (SCMR) guidance for the practice of cardiovascular magnetic resonance during the COVID-19 pandemic. J Cardiovasc Magn Reson.

[78] Beitzke D, Salgado R, Francone M (2020). Cardiac imaging procedures and the COVID-19 pandemic: recommendations of the European Society of Cardiovascular Radiology (ESCR). Int J Cardiovasc Imaging.

[79] Galea N, Catapano F, Marchitelli L (2020). How to perform a Cardio-thoracic MRI in COVID-19: comprehensive assessment of heart, pulmonary arteries and lung parenchyma. Eur Hear J Cardiovasc Imaging.

